# Rhoifolin: A promising flavonoid with potent cytotoxic and anticancer properties: molecular mechanisms and therapeutic potential

**DOI:** 10.17179/excli2024-7836

**Published:** 2025-02-25

**Authors:** Ceyda Sibel Kiliç, Mehmet Murat Kisla, Gülin Amasya, Ceyda Tugba Sengel-Türk, Zeynep Ates Alagöz, Ayse Mine Gençler Özkan, Ilker Ates, Safa Gümüsok, Jesús Herrera-Bravo, Javad Sharifi-Rad, Daniela Calina

**Affiliations:** 1 Department of Pharmaceutical Botany, Faculty of Pharmacy, Ankara University, Tandogan, Türkiye; 2 Department of Pharmaceutical Chemistry, Faculty of Pharmacy, Ankara University, Tandogan, Türkiye; 3 Department of Pharmaceutical Technology, Faculty of Pharmacy, Ankara University, Tandogan, Türkiye; 4 Department of Pharmaceutical Toxicology, Faculty of Pharmacy, Ankara University,Tandogan, Türkiye; 5 Departamento de Ciencias Básicas, Facultad de Ciencias, Universidad Santo Tomas, Santiago, Chile; 6 Universidad Espíritu Santo, Samborondón 092301, Ecuador; 7 Department of Medicine, College of Medicine, Korea University, Seoul 02841, Republic of Korea; 8 Department of Clinical Pharmacy, University of Medicine and Pharmacy of Craiova, 200349 Craiova, Romania

**Keywords:** rhoifolin, apigenin-7-O-neohesperidoside, flavonoid, Citrus, anti-cancer activity

## Abstract

Rhoifolin is a flavonoid found in various plant species, especially within the Rutaceae family, and is considered a dietary component due to its presence in edible plants. Its bioactive properties, such as cytotoxic and anticancer activities, have gained significant attention. This review aims to highlight the general properties and diverse bioactivities of rhoifolin, with a particular focus on its cytotoxic and anticancer effects. This is based on a comprehensive literature search, focusing on the presence of rhoifolin in different plant species and its biological activities, particularly its anticancer properties. Rhoifolin is widely distributed in the plant kingdom, especially in *Citrus* species. It exhibits a variety of bioactivities, including strong cytotoxic and anticancer effects. Recent studies have shown that rhoifolin can induce apoptosis and inhibit cancer cell proliferation, making it a promising candidate for anticancer therapies. Rhoifolin's diverse bioactivities, particularly its cytotoxic and anticancer properties, position it as a potential therapeutic agent. Further detailed investigations into its molecular mechanisms and well-designed clinical studies are needed to fully understand and utilize its therapeutic potential.

See also the graphical abstract(Fig. 1).

## Introduction

Flavonoids, a group of omnipresent plant metabolites, have been recognized as phytochemicals with specific pharmacological activities associated with human health. The therapeutic efficacy of fully characterized flavonoids such as quercetin, curcumin, berberin, rutin, apigenin, naringenin, catechin etc. on various chronic diseases, especially cancer, has been demonstrated in many studies (Rasouli et al., 2019[145]). Flavonoids are important secondary metabolites that are known to be present in fruits and vegetables, having a wide spectrum of bioactivities like antiviral, antiplatelet, anti-allergic, antioxidant, anti-inflammatory, hepatoprotective, insulin mimetic, protective in cardiovascular diseases, antitumor and highly selective cytotoxic activities that might be beneficial for human health (Tanwar and Modgil, 2012[175]; Refaat et al., 2015[147]). However, the safe use of herbal medicines poses a significant barrier to their development due to insufficient data on the incidence of side effects (Dores et al., 2023[41]). Numerous studies have been carried out in the recent past related to the anti-proliferative activities of flavonoids in general. Furthermore, numerous studies focused on the anticancer activity of *Citrus* flavonoids, *Citrus *juices and extracts, in which rhoifolin was also found to be present (Benavente-Garcia and Castillo, 2008[25]; Visalli et al., 2014[182]; Song et al., 2022[166]). *Citrus aurantium* L. (bitter orange) is among these species having rhoifolin and also having anti-cancer usage (Suryawanshi, 2011[172]). There are other plants with cytotoxic and/or anti-cancer activity having this important secondary metabolite within their compositions, as well (Hasibuan et al., 2020[69]; Persia et al., 2020[134]). Thus, this review is planned to focus on the anti-cancer effect of rhoifolin which is found to be present in different plant species belonging to different families. Rhoifolin is a flavone glycoside that is also known as apigenin-7-*O*-neohesperidoside (Lefort and Blay, 2011[96]), isolated firstly from fresh *Rhus succedanea *L. leaves, a plant species belonging to the Anacardiaceae family (Hattori et al., 1952[70]; Huang et al., 2014[75]). One of the earliest records related to rhoifolin that is found in many different plant species, dates back to 1952. When the compound was first isolated, it was reported to be a new flavone glycoside of apigenin (apigenin-7-rhamnoglucoside) (Hattori et al., 1952[70]), however in some studies, the compound is named as apigenin-7-*O*-neohesperidoside, as well (Hafez et al., 2003[66]; Abd Elhameid et al., 2006[2]). When we look at the plant kingdom, various *Citrus *plants are known to possess this compound in general, such as oranges, bergamot, lemon, kumquat, mandarin, tantor etc. (Ramful et al., 2010[143]; Refaat et al., 2015[147]). 

## Methodology

A comprehensive literature search was conducted to identify relevant studies on the anticancer properties of rhoifolin. The search was performed across multiple electronic databases, including PubMed/MedLine, Scopus, Web of Science, and Google Scholar. The search strategy involved the use of Medical Subject Headings (MeSH) terms and Boolean operators to ensure a thorough and precise retrieval of relevant articles. The primary MeSH terms used included "r," "Flavonoids," "Antineoplastic Agents," "Cytotoxicity," and "Nanoparticles." These terms were combined using Boolean operators such as "AND," "OR," and "NOT" to refine the search. For example, the search query "rhoifolin AND (Antineoplastic Agents OR Cytotoxicity) AND Nanoparticles" was used to capture studies focusing on the anticancer properties of rhoifolin and its delivery via nanoparticles. The search was limited to articles published in English. Studies were included if they were original research articles, reviews, or meta-analyses focusing on the anticancer properties of rhoifolin. Both *in vitro* and *in vivo* models were considered. Studies were required to report on the anticancer activity of rhoifolin, including its cytotoxic effects, mechanisms of action, and potential for clinical application. No restrictions were placed on the publication date, allowing the inclusion of both historical and recent studies. Studies that were not peer-reviewed, such as conference abstracts, editorials, or opinion pieces, were excluded. Articles that did not specifically address the anticancer properties of rhoifolin or focused on unrelated bioactivities were excluded. Additionally, articles published in languages other than English were excluded. Data from the selected studies were extracted independently by two researchers using a standardized data extraction form. The extracted data included study design, model systems used (e.g., cell lines, animal models), outcomes measured (e.g., cell viability, apoptosis induction, tumor growth inhibition), and key findings related to the anticancer properties of rhoifolin. The most important data were synthesized and summarized in tables and figures to provide a clear and concise overview of the findings. These visual aids include details on the types of cancer cells affected by rhoifolin, its mechanisms of action, and the efficacy of different delivery systems, particularly nanoparticle-based approaches. Furthermore, the chemical structures and taxonomy of the plants associated with rhoifolin were validated using PubChem and the World Flora Online (WFO) database to ensure accuracy and consistency in the scientific details reported (PubChem[124], WFO, 2023[189]).

## Rhoifolin: a Brief Overview

### Natural sources 

When we search the literature, we can see that this compound is quite common in the nature and is present in many species of different plant families. A tabulated list of some of these plants is presented in alphabetical order in Table 1(Tab. 1) (References in Table 1: Abbas et al., 2021[1]; Abd Elhameid et al., 2006[2]; Abiri et al., 2021[3]; Agogbua et al., 2022[6]; Alfarisi et al., 2020[8]; Alreshidi et al., 2020[9]; Amoroso et al., 2021[12]; Andrade-Pinheiro et al., 2023[13]; Awad et al., 2014[15]; Barberis et al., 2020[19]; Barreca et al., 2011[22][21][23]; Bindu and Udayan, 2018[26]; Brinza et al., 2020[27]; Burlando et al., 2017[30]; Cai et al., 2020[31]; Casabuono and Pomilio, 1990[32]; Chen et al., 2021[35], 2023[34]; Cheng et al., 2017[36]; Cicero et al., 2017[37]; Dai et al., 2021[38]; Dhanabal et al., 2006[40]; Dormousoglou et al., 2023[42]; Egger and Keil, 1969[43]; Ekeke et al., 2019[44]; Eldahshan and Azab, 2012[46]; Eldahshan, 2013[45]; Elma et al., 2019[47]; El-Shawi and Eldahshan, 2014[48]; El-Shawy, 2014[49]; Eroğlu Özkan et al., 2019[50]; Fahim et al., 2015[52]; Fan et al., 2019[53]; Fenglin et al., 2004[54]; Gattuso et al., 2006[57]; Ge, 2014[58]; Giambanelli et al., 2018[60]; Guetchueng et al., 2020[65]; Hafez et al., 2003[66]; Han et al., 2023[67]; Hasibuan et al., 2020[69]; Hattori et al., 1952[70]; He et al., 2003[71]; Hong et al., 2021[73]; Huang et al., 2014[75]; Huo et al., 2021[77]; Huynh et al., 2016[78]; Jaiswal et al., 2019[80]; Juee, 2022[83]; Kanao and Matsuda, 1978[84]; Kaneko et al., 1995[85]; Kanes et al., 1993[86]; Kayahan and Saloğlu, 2022[88]; Kiem et al., 2010[90]; Kumar et al., 1999[93]; Kuo et al., 2017[94]; Lee et al., 2010[95], Liang et al., 2007[97]; Lin et al., 1994[100], 1998[99], 2023[101]; Liu et al., 2012[105], 2013[102], 2021[103], 2023[106]; Lopez-Gutierrez et al., 2015[109], 2016[110]; Ma et al., 2013[113], 2014[114], 2018[112]; Mencherini et al., 2013[122]; Nakajima et al., 2014[123]; Negm et al., 2022[125]; Neretina et al., 2005[126]; Okonwu and Muonekwu, 2019[129]; Ozkan et al., 2022[130]; Papalia et al., 2017[131]; Pernice et al., 2009[133]; Persia et al., 2020[134]; Plioukas et al., 2016[135]; Prashar and Patel, 2020[137]; Qiaoyu and Lingsheng, 1996[140]; Rahmouni et al., 2022[141]; Rajkumar and Jebanesan, 2008[142]; Rao et al., 2011[144]; Sawikowska, 2020[150]; Saxena et al., 2014[152], 2016[151]; Sayeed et al., 2023[153]; Senizza et al., 2021[154]; Seukep et al., 2020[155]; Sharifi-Rad et al., 2021[156]; Shen et al., 2017[158]; Shimokoriyama, 1966[159]; Son et al., 1992[165]; Song et al., 2022[166]; Souilah et al., 2020[169], 2021[168]; Sultana et al., 2018[171]; Suryawanshi, 2011[172]; Taheri et al., 2023[173]; Tsujimoto et al., 2019[177]; Uddin et al., 2011[178]; Uysal et al., 2019[179]; Vega-Ruiz et al., 2021[181]; Visalli et al., 2014[182]; Vo et al., 2022[183]; Wang et al., 2010[187], 2016[185], 2017[184]; Xie et al., 2021[191]; Yadav et al., 2009[193]; Yang et al., 2021[195]; Ye et al., 2014[197]; Yıldız Turgut et al., 2019[198]; Zengin et al., 2021[199]; Zhang et al., 2005[201], 2009[200], 2022[202]; Zhang, 2007[204]; Zheng et al., 2022[205]; Zhou et al., 2023[207][208]). In time, taxonomical hierarchy and nomenclature of some of these species have changed; therefore, synonyms for these species (if present) are also provided.

Today, biotechnological studies on plants use plant *in vitro* culture strategies to produce therapeutically effective phytochemicals with a completely safe efficacy profile without metabolite variations due to different geographical and climatic conditions (Thorpe, 2007[176]; Khan et al., 2021[89]). Although *in vitro* culture technologies have an increasing interest and importance due to their potential to grow any plant anywhere, to provide a sophisticated production platform for phytochemicals, and to enhance new plant-based medicinal compounds, there has been still no biotechnological-based *in vitro* cell culture study for the production of rhoifolin in the literature.

### Chemical characterization 

When the importance of flavonoids that constitute a diverse group of polyphenolic compounds having various and significant biological activities including antiinflammatory, cardioprotective, antidiabetic, and anti-cancer effects were understood, many researchers started to investigate their health-related benefits (Nijveldt et al., 2001[127], Refaat et al., 2015[147]). Rhoifolin (apigenin 7-*O*-neohesperidoside) is a flavonoid and an apigenin derivative that bears an alpha-(1->2)-L-rhamnopyranosyl)-beta-D-glucopyranosyl moiety that is attached to the 7-hydroxy group. Several studies on the diverse activity potentials of the mixtures having this compound have been reported in the literature (Lou et al., 2016[111]; Burlando et al., 2017[30]; Kuo et al., 2017[94]; Brinza et al., 2020[27]; Seukep et al., 2020[155]; Qi and Liu, 2022[139]; Zheng et al., 2022[206]). The chemical structure, nomenclature, and spectroscopic properties of rhoifolin are given in Table 2(Tab. 2). In respect to chemical nomenclature, rhoifolin is apigenin 7-*O*-*β*-neohesperidoside having C_27_H_30_O_14_ chemical formula that has a molecular weight of 578.53 (exact mass: 578.1636). The compound is usually isolated as yellow amorphous powder or yellow needles after crystallization from methanol (melting point: 245-253 °C). It is soluble in hot ethanol, methanol, water; sparingly soluble in cold ethanol, ethyl acetate, and insoluble in chloroform and *n*-hexane (Refaat et al., 2015[146]).

### Semi-synthetic derivatives

Studies regarding the derivatization of rhoifolin are limited in the literature. One of them is the transglycosylation route conducted by Aoki et al., in which alpha-glucosyl rhoifolin was synthesized and its ^1^H and ^13^C-NMR signals were identified by using a variety of NMR methods (HSQC, COSY, HMBC, and 1D TOCSY) and mass spectrometry. Accordingly, the assignment of the signals for rhoifolin and sugar moieties was completed. With the help of correlations in the HMBC spectra, it was understood that intramolecular hydrogen bonds were present within the flavone and the flavonol skeletons of Rhf-G, as shown in Figure 2(Fig. 2) (Aoki et al., 2017[14]).

The usage of rhoifolin as an intermediate was a strategy implemented for the chemo-enzymatic synthesis of acacetin (Hanamura et al., 2016[68]). To achieve the end product, rhoifolin (**1b**) was prepared from naringin (**3a**), which is essentially an oxidation process with I_2_ and pyridine at 95 ℃. After cooling down, forming **1b **was acetylated by adding acetic anhydride into the mixture. Then, this mixture was heated for 6 h at 95 ℃ to yield **1c** using the process given in the literature. For the enzymatic deacetylation of R_2_, *Candida antarctica *lipase B was used at given conditions to afford **1d **with excellent yield (98 %). An *in-situ* formed diazomethane solution was used to methylate **1d **at the same position, to achieve **1e**. To prepare this solution beforehand, *N-*methyl-*N***-**nitroso-*p*-toluene-sulfonamide was added to the solution of KOH in water and EtOH. For the total deacetylation of **1e** to obtain **1f**, this compound was dissolved in methanol and added to a solution of sodium methoxide. Finally, sugar side chain of **1f **was cleaved to obtain **1a **with high yield and regioselectivity, using concentrated H_2_SO_4_ and a reflux set-up (Figure 3(Fig. 3); Reference in Figure 3: Hanamura et al., 2016[68]). 

## Anticancer Activities of Rhoifolin

### Mechanism of antitumor action of rhoifolin 

**Figure 1:**
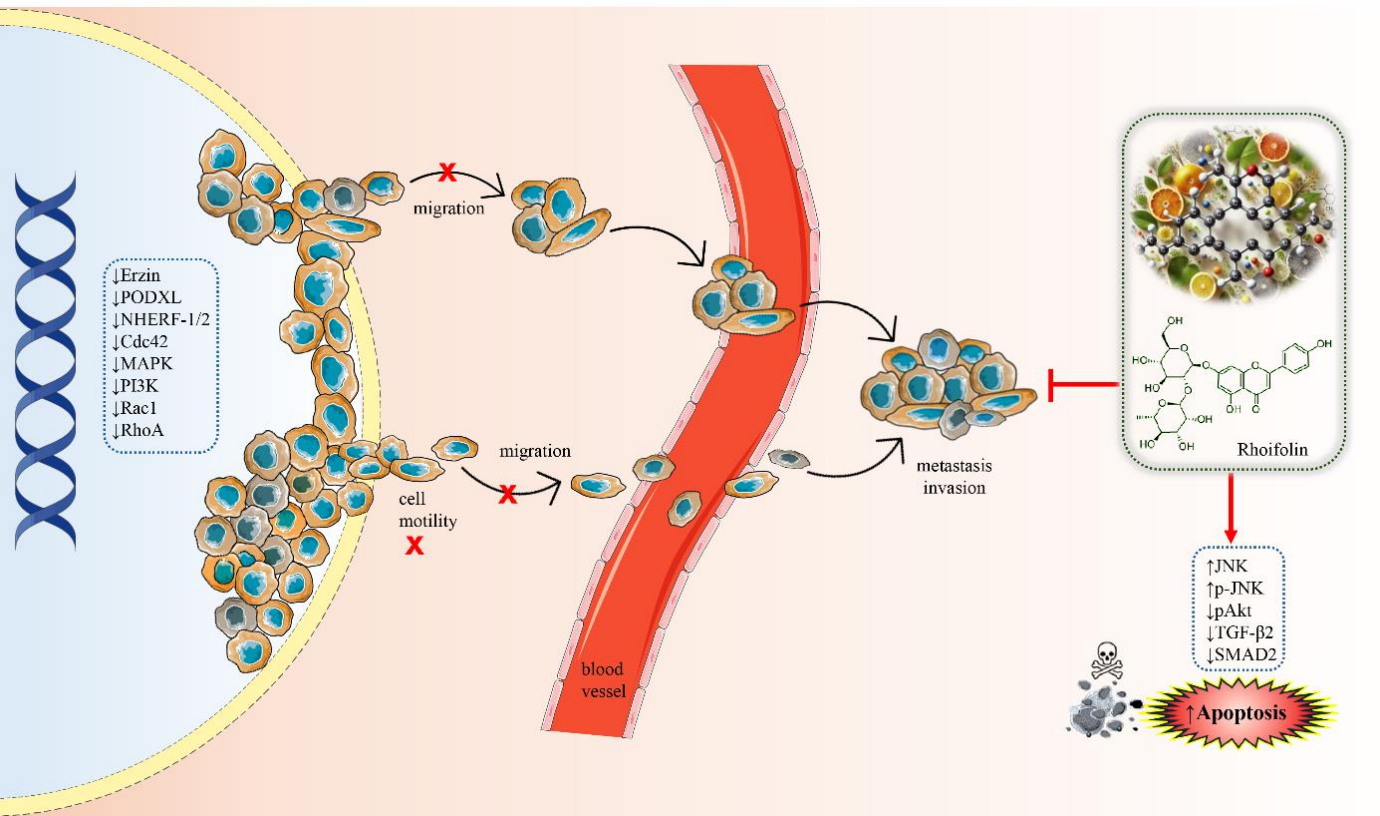
Graphical abstract: Mechanism of rhoifolin's anti-metastatic and pro-apoptotic effects on cancer cells. This diagram illustrates the anti-metastatic and pro-apoptotic effects of rhoifolin on cancer cells. The left side of the image shows the inhibition (↓) of key signaling molecules such as ezrin, PODXL, NHERF-1/2, Cdc42, MAPK, PI3K, Rac1, and RhoA, which are involved in cell motility and migration. This inhibition prevents the migration of cancer cells from the primary tumor site and their entry into the bloodstream, effectively reducing metastasis and invasion. The right side of the image depicts the chemical structure of rhoifolin and its role in promoting apoptosis (↑Apoptosis) by modulating various molecular pathways, including the upregulation (↑) of JNK and p-JNK, and the downregulation (↓) of pAkt, TGF-β2, and SMAD2, leading to increased cancer cell death. Abbreviations: Cdc42, cell division control protein 42 homolog; JNK, c-Jun N-terminal kinase; MAPK, mitogen-activated protein kinase; NHERF, Na+/H+ exchanger regulatory factor; PI3K, phosphoinositide 3-kinase; PODXL, podocalyxin-like protein; Rac1, Ras-related C3 botulinum toxin substrate 1; RhoA, Ras homolog family member A; SMAD2, mothers against decapentaplegic homolog 2; TGF-β2, transforming growth factor-beta 2)

**Figure 2:**
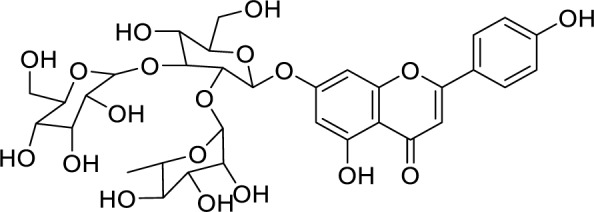
Chemical structure of Rhf-G

**Figure 3:**
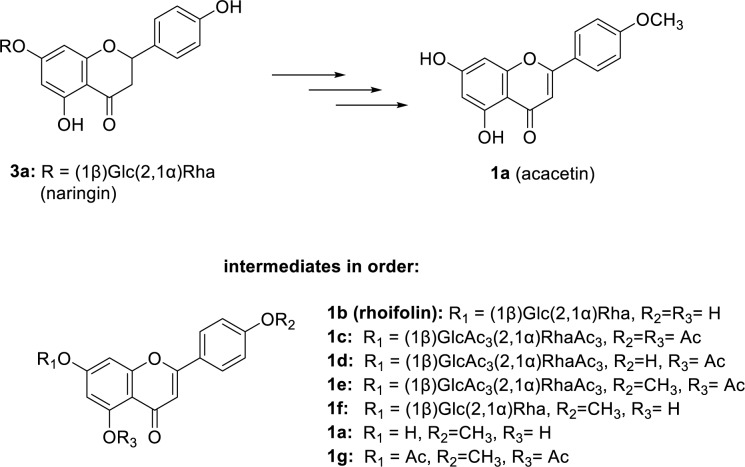
Synthetic route for 1a (acacetin) from 3a (naringin) via compounds 1b-1g. Chemical structures for these compounds were also given (adapted from Hanamura et al., 2016)

**Figure 4:**
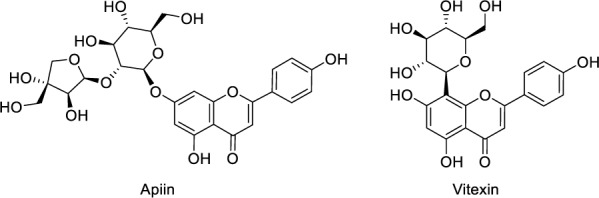
Chemical structures of apiin and vitexin

Rhoifolin is present in many plant species and one of them is *Callicarpa nudiflora* Hook & Arn. which grows widely in southern China. Xiong and colleagues (2021[192]) investigated the anti-motile effects of rhoifolin in this plant, to evaluate if this effect on cell motility would produce anti-cancer effect. Ezrin and podocalyxin (PODXL) are the main actors that would organize membrane proteins and signal transduction mechanisms, thus eventually modulating the cytoskeleton rearrangement in cell motility.The latter regulates cell motility by interacting with actin polymerization complex composed of ezrin, and PDZ proteins NA+/H+ exchanger regulatory factor isoforms 1 and 2 (NHERF-1/2) (Sizemore et al., 2007[163]). Ezrin and NHERF-1/2 are adaptor proteins facilitating the interaction of PODXL with the cytoskeleton within epithelial cells. PODXL, ezrin, and NHERF-1/2 interactions result in metastasis induction via Cdc42, MAPK, PI3K, Rac1, and RhoA (McNagny et al., 2012[120]; Flores-Tellez et al., 2015[56]). RFL's activity on breast cancer cell viability was assessed via MTT assay. Antimigratory properties were elucidated by ORISTM cell migration assay and immuno-precipitation was performed to assessing the effects of RFL on the interaction between ezrin and PODX. Results demonstrated that RFL resulted in remarkable inhibitions on cell migration and alterations in the location and organization of actin cytoskeleton in breast cancer cells. Secondly, RFL suppressed ezrin phosphorylation and its interaction with PODXL, thus exerting anti-motile effect. Moreover, it inhibited TGF-β1-induced EMT in MDA-MB-231 cells. As a conclusion, it was deduced that the anti-motile action of RFL was due to its potential downregulatory effect on PODXL-Ezrin interaction during EMT (Xiong et al., 2021[192]). In another study, Zheng and colleagues investigated the potential anticancer effects of flavonoids in *Plumula nelumbinis*, which is the green embryo of the plant named *Nelumbo nucifera *Gaertn. against pancreas cancer. According to high-performance liquid chromatography (HPLC) and mass spectrometry (MS) results, rhoifolin, apiin, and vitexin were found to be the most abundant compounds (Figure 4(Fig. 4) and Figure 1(Fig. 1): Graphical abstract). Cell viability tests showed that these three compounds have the potential to inhibit PANC-1 and ASPC-1 cell lines' proliferation. Among these compounds, rhoifolin was the most potent compound, which also promoted apoptosis of pancreatic cancer cells via up-regulation of JNK and p-JNK with the down-regulation of p-Akt. Furthermore, this compound led to the inhibition of cell migration and invasion while it effectively enhanced antioxidant capacity of PANC-1 and ASPC-1. AKT activator (SC79) and JNK inhibitor (SP600125) that both reversed the anticancer effects of rhoifolin on pancreatic cancer cells, thus validating the mechanism of this compound. According to quantitative proteomics analysis, it also modified proteomic profiles in these cancer cell lines. Western blot analysis results demonstrated the downregulation of transforming growth factor beta 2 (TGF-β2) and phosphorylated SMAD family member (SMAD2). All these findings suggest that rhoifolin may exert its anti-pancreatic cancer activities through signaling pathways of Akt/JNK/ caspase-3 and TGF-β2/SMAD2 (Zheng et al., 2022[205]).

### Evidence from preclinical studies confirmed the anticancer properties 

**Figure 5:**
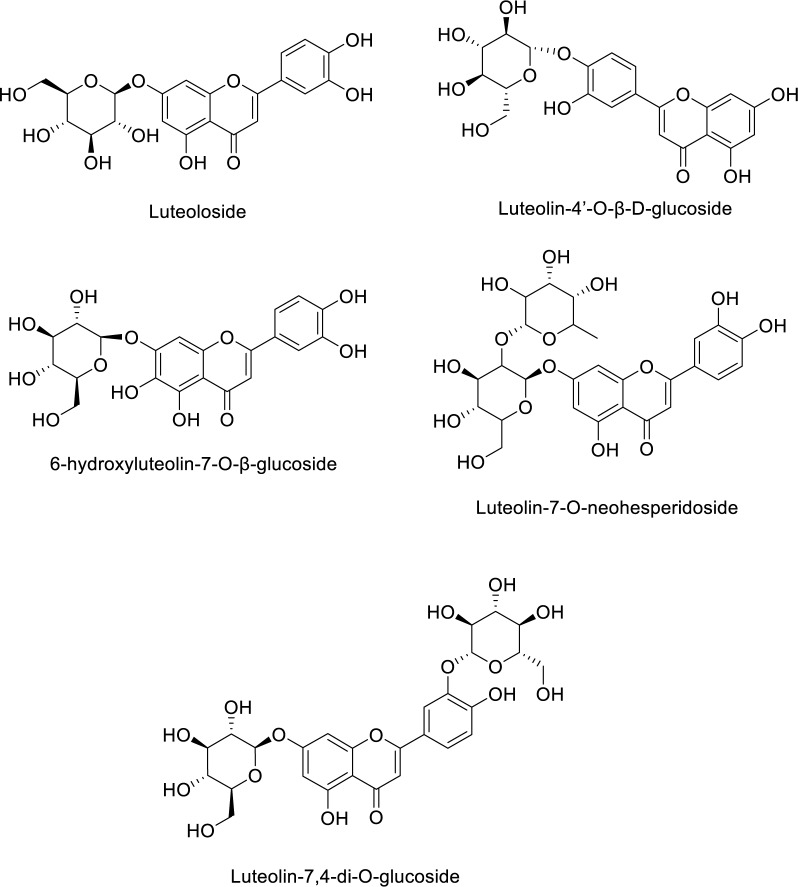
Chemical structures of the compounds mentioned in Ma et al. (2014a)

Anticancer action of rhoifolin in the plant species has been evidenced by a few assays according to the existing literature (Table 3(Tab. 3); References in Table 3: Eldahshan, 2013[45]; Koch et al., 2005[91]; Ma et al., 2014[115]; Xiong et al., 2021[192]; Zheng et al., 2022[205]). For instance, Ma and co-workers assessed the cytotoxicity of the EtOH extracts prepared from *Callicarpa nudiflora *by the MTT assay. The flavonoid composition of this plant was isolated and purified by HP-20 macroporous resin, silica gel and Sephadex LH-20 column chromatography methods. The structures of the twelve isolated compounds were elucidated by spectroscopic data and one of them was identified as rhoifolin. Among these compounds, luteoloside, luteolin-4'-O-β-D-glucoside, 6-hydroxyluteolin-7-O-β-glucoside, luteolin-7-O-neohesperidoside, rhoifolin, luteolin-7,4-di-O-glucoside (Figure 5(Fig. 5)) exerted proliferation inhibitory activities, in various concentrations, against HeLa, A549, and MCF-7 cells. Meanwhile, 6-hydroxyluteolin-7-O-β-glucoside, rhoifolin, and nudifloside produced much higher cytotoxic activities (Ma et al., 2014[115]).

Eldahshan studied the antitumor activity of rhoifolin against human epidermoid larynx (Hep 2), human cervical (HeLa), hepatocellular (HepG2), colon (HCT-116) and fetal human lung fibroblast (MRC-5) carcinoma cell lines. According to the assay results, the compound exhibited high cytotoxicity against Hep 2 with IC_50_=5.9 μg/mL whereas the value for the standard vinblastine was 4.6 μg/mL. The IC_50_ value for HeLa was 6.2 μg/mL and for vinblastine it was 5.2 μg/mL. The IC_50_ of rhoifolin was 22.6 μg/mL for HepG2, 34.8 μg/mL for HCT-116 and 44 μg/mL for MRC-5. Moreover, calculated selectivity index (SI) data for rhoifolin was greater than 8.47 for Hep 2, followed by 8.06 for HeLa and 2.21 for HepG2.

The compound was found to be safe for these cells, and toxic for colon and fetal human lung fibroblast cell lines, since an SI value less than 2 indicates the general toxicity of the compound (Koch et al., 2005[91]; Eldahshan, 2013[45]).

## Other Biological Activities of Rhoifolin

Today, the tendency to use natural and traditional medicines to cope with chronic diseases that have difficulties in diagnosis and treatment has increased (Sharma et al., 2024[157]). In this context, it is essential to consider rhoifolin and other natural bioactive compounds in terms of their scientific, medical, and traditional applications, particularly given the growing interest in flavonoid chemical structures and their pharmacological properties. The literature search result showed that this compound is widely distributed in many families of the plant kingdom and can be obtained in significant quantities especially from *Citrus* and *Chorisia *species. Moreover, *in vitro* and *in vivo* studies have shown that rhoifolin has many biological activities. According to these pharmacological findings, rhoifolin is on its way to being among the most preferred drugs, especially due to its strong anti-inflammatory, hepatoprotective, hypoglycemic and highly selective cytotoxic effects. Therefore, in the future, a detailed investigation of the molecular mechanisms of these activities will be necessary, along with well-designed clinical studies (Eldahshan, 2013[45]; Refaat et al., 2015[146]).

### Antioxidant properties

**Figure 6:**
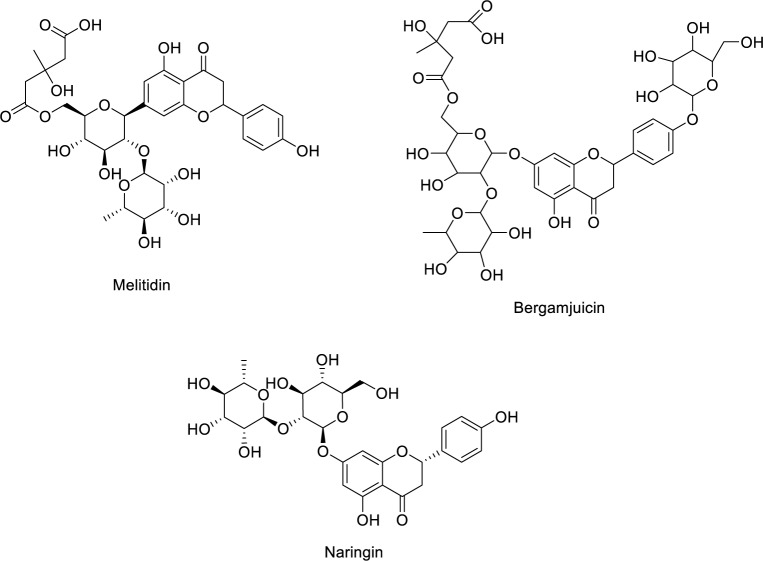
Chemical structure of melitidin, bergamjuicin, and naringin

The dynamic interplay of oxidative stress, cellular communication and signaling pathways is fundamental to understanding the initiation, progression, and therapeutic resistance of cancer (Alshehri et al., 2022[11]; Iqbal et al., 2024[79]). Since the health benefits of flavonoids are accredited to their antioxidant activities, *Citrus grandis *L. Osbeck (Shatianyu) was investigated by Mei Deng and co-workers for its chemical composition of flavonoids. In their study, they isolated and identified 11 flavonoids from Shatianyu pulp flavonoid extracts (SPFEs). They also evaluated the cellular antioxidant activity (CAA) and oxygen radical absorbance capacity (ORAC) of the isolated compounds. Naringin and rhoifolin showed the highest ORAC activities while SAR results suggested that 3-hydroxy-3-methylglutaryl or 4'-glucose decreases the ORAC activity of flavonoids. The contribution to the holistic antioxidant activity of these flavonoids was evaluated by an online knockout method in Table 4(Tab. 4) (Reference in Table 4: Deng et al., 2022[39]). ORAC activity was mostly influenced by melitidin, bergamjuicin and naringin (Figure 6(Fig. 6)) (Deng et al., 2022[39]). 

**Figure 7:**
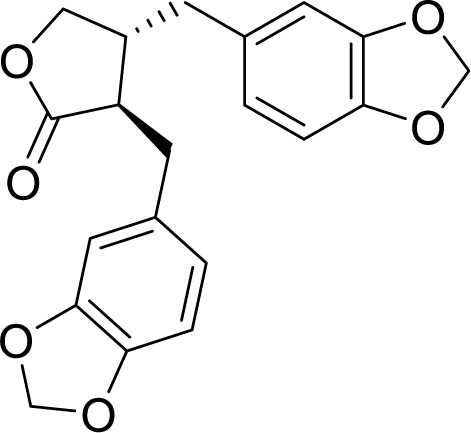
Chemical structure of hinokinin

Another example of ORAC analysis to determine antioxidant activity was conducted by Zhang et al. In their study, they separated and identified the components of *Piper nigrum* L. leaf and fruit extracts. According to the obtained results, the ORAC value of leaf extracts was 3639.05 μmol TE/g, which was greater than that of the fruit extracts. Among all the solvents, the ethanol extract was determined to exert the highest ORAC value. Consequently, active components were isolated from ethanol extracts of *Piper nigrum* leaves with silica gel chromatography (Sephadex LH-20 resin), reversed-phase chromatography, gel-filtration chromatography, thin-layer chromatography and HPLC. Structural confirmations of hinokinin (Figure 7(Fig. 7)) and rhoifolin were performed by utilizing nuclear magnetic resonance spectroscopy (NMR) and mass spectrometry (MS) techniques. ORAC values for these compounds were found to be 16070 μmol TE/g and 10823 μmol TE/g, respectively (Zhang et al., 2015[203]).

Wang and co-workers evaluated the DPPH radical scavenging activity of the *n*-butanol extract of *Lonicera japonica* Thunb. leaves. In this assay, they assessed the extract and the compounds which were identified with HPLC present in the extract. Ascorbic acid and Vitamin E were used as standards (Table 3(Tab. 3)). Authors concluded that this crude drug had an antioxidant potential value (11.2 µg/mL) close to that of Vitamin E (9.5 µg/mL) (Table 5(Tab. 5); Reference in Table 5: Wang et al., 2017[184]). Moreover, they isolated the compounds in the extract and also identified their DPPH scavenging activities. Based on the table below, compounds **1-9** (rhoifolin being **4**) exhibited high radical scavenging activity which eventually translate to high antioxidant activity (Wang et al., 2017[184]).

### Anti-inflammatory effect

Inflammation, arising as local response of living mammalian tissues when injured, is also a fundamental protective process that aims to preserve the general structure of the organism against infectious, physical and chemical attacks (Eldahshan and Azab, 2012[46]). However, when the body is exposed to toxins and other agents like chronic stress, obesity, and autoimmune disorders, unwanted body responses can sometimes be triggered. In this case, instead of curing the problem and then restoring the normal functioning, inflammation might persist; proinflammatory cytokines, chemokines, adhesion molecules and inflammatory enzymes come into play. Today, it is thought that this chronic inflammatory condition can result in various health problems, including arthritis, depresssion, cardiovascular problems, Alzheimer's Disease (AD) and even cancer (Singh et al., 2019[161]). Steroids, nonsteroidal anti-inflammatory drugs (NSAIDs) and immunosuppressants that are used to control and/or suppress the inflammatory crisis might result in various side effects, as well. In practice, the aim is to administer the minimum effective dose with the highest efficiency and the fewest side effects. Therefore, it is necessary to incorporate natural anti-inflammatory factors in drug therapy to achieve an enhanced pharmacological response, and the lowest extent of adverse effects. In this context, herbal medicines are prominent agents in medicine (Ghasemian et al., 2016[59]). Throughout history, various herbs have been used in the treatment of inflammation and associated ailments like rheumatism all over the world. Flavonoids found in plants used in many traditional medicines have been associated with this activity. In this context, apigenin, a phytopolyphenol commonly found in the human diet, is an important example. It was confirmed by Sawatzky et al. that apigenin, like many other flavonoids, exerts anti-inflammatory effects like reducing oxidative stress and preventing the expression of various inflammatory factors (Sawatzky et al., 2006[149]). With the knowledge provided by the apigenin glycoside study; a different study group was motivated to investigate rhoifolin's anti-inflammatory activity that has not been tested previously. Rhoifolin administered at doses of 2.5, 25, and 250 mg/kg resulted in a significant inhibition of rat paw edema of 14 %, 25 %, and 45 %, respectively, after 4 hours of treatment compared to the control group (74 %). In addition to the significant abolition of prostaglandin E2 levels with increasing rhoifolin doses, TNF-α release in inflammatory exudates was significantly reduced. In this study, rhoifolin was found to have potent anti-inflammatory activity at low doses (Eldahshan and Azab, 2012[46]). The study by Peng et al. (2020[132]) aimed to reveal the effect of rhoifolin on arthritis induced by complete Freund's adjuvant (CFA) in rat models. Significant improvement was observed in paw edema and weight loss parameters with the administration of Rholifolin at doses of 10 and 20 mg/kg. These improvements were also confirmed with the data obtained as a result of histopathological observations. Additionally, significant decrease in oxidative stress was observed with rhoifolin administration, as evidenced by the changes in intracellular glutathione, glutathione peroxidase, superoxide dismutase and malondialdehyde levels in the tissue of articular cartilage. Besides, proinflammatory cytokines, tumor necrosis factor (TNF)-α, interleukin (IL)-1β and IL-6 gene expression exhibited a significant downregulation of gene expression. According to this group, antioxidant and anti-inflammatory effects of the compound were probably via the NF-κB pathway, however, the exact compounds responsible for this action need to be determined in future studies. Osteoarthritis (OA) or degenerative arthritis, which reduces the quality of life of approximately 250 million people worldwide, is an important public health problem today. It is a chronic musculoskeletal disease, especially seen in the elderly population and affects mobile connections between two bones such as the knee and hip joints (Kraus et al., 2015[92]). OA affects all structures of the joints and is characterized by cartilage destruction, remodeling of subchondral bone, osteophyte formation, and changes in the synovium and joint capsule (Goldring and Goldring, 2010[61]). In some studies, it has been shown that proinflammatory cytokines can trigger cartilage destruction by directing the production of catabolic degrading enzymes in chondrocytes. Therefore, inhibition of synovial joint inflammation might be an effective form of treatment for OA (Meliconi and Pulsatelli, 2019[121]). A study based on this information aimed to address the protective effects of rhoifolin on OA with some *in vitro* and *in vivo* experiments. Results showed that rhoifolin suppressed senescence-associated secretory phenotype factors' expression and the senescence phenotype in IL-1β-treated chondrocytes. Additionally, rhoifolin inhibited IL-1β-induced activation of the NF-κB pathway. Molecular docking and knock-down studies demonstrated that rhoifolin might also bind to Nrf2 to suppress the NF-κB pathway. Finally, rhoifolin was shown to ameliorate the OA process in an *in vivo* ACLT rat model (Chen et al., 2022[33]). Another study conducted by Yan et al. also contributed to the knowledge of the therapeutic effects of rhoifolin and provided a new perspective for prospective treatment of OA. They clearly reported the anti-inflammatory, anti-cartilage degradation and autophagy promoting properties of rhoifolin, which was confirmed to function by regulating autophagy. Furthermore, P38/JNK and PI3K/AKT/ mTOR pathways were also involved in the process (Yan et al., 2021[194]).

### Neuroprotective 

Neurodegeneration is a complication of incurable age-related diseases that have a toll on the nervous system and significantly reduces the life qualities of both elderly patients and their families (Azzini et al., 2024[16]). With the prolongation of life expectancy in modern societies, the prevalence of neurodegenerative diseases has also increased, and this issue has turned into a globally recognized public health problem and it is well-known that one of the most important causes of age-related dementia is AD. Today, there is no effective treatment for this disease. Recently, nutrition has been considered a very important factor in the protection of the body against chronic inflammation and oxidative stress that lead to chronic degenerative diseases (Ezzat et al., 2024[51]). Numerous bioactive food components might affect the pathological mechanisms underlying AD. Among these, phenolic compounds, omega-3 fatty acids, isothiocyanates, fat-soluble vitamins and carotenoids are promising agents in this respect (Grodzicki and Dziendzikowska, 2020[63]). In a study, ameliorative effects of rhoifolin on zebrafish anxiety induced by scopolamine, amnesia, brain oxidative stress and the mechanisms underlying these disorders were investigated. For nine consecutive days, rhoifolin (1, 3 and 5 μg/L) and then scopolamine (100 μM) were administered to Zebrafish 30 minutes before behavioral tests (novel tank diving test, Y-maze and novel object recognition tests). Rhoifolin, isolated from the leaves of *Chorisia crispiflora* Kunth (Malvaceae) can alleviate memory deficits, anxiety, brain oxidative stress in scopolamine-treated zebrafish and regulate cholinergic function by inhibiting AChE activity. The results of this study showed that rhoifolin was a promising compound against amnesia and anxiety via restoration of cholinergic activity and improving brain oxidative stress (Brinza et al., 2020[27]). Spinal cord injury usually results from physical damage that results in infiltration of inflammatory cells and secondary degeneration. It leads to detrimental impairment of neurological dysfunctions (autonomic, motor and sensory) (Ahn et al., 2015[7]). In a study designed with the thought that the widely known antioxidative property of rhoifolin may be effective in spinal cord injury, rhoifolin inhibited proinflammatory cytokines and NF-κB pathways, and significantly reduced intracellular oxidative stress in the spinal cord-injured rat model. Overall, there were significant improvements in motor function in rats treated with rhoifolin. Thus, the study evidently confirmed that rhoifolin could be a safer alternative than current treatments for spinal cord injury. It has been reported that the effect may be due to a decrease in apoptotic signal, as observed in reduced levels of p38MAPK and caspases (Long et al., 2023[108]). Rhoifolin was also investigated in the experimental rat model of AD, induced by streptozotocin administration. Rhoifolin's effect on the thickness of the CA1 pyramidal layer and spatial learning in the AD model were examined, as well. The Morris water maze test and novel object recognition test were used to demonstrate the effects on relieving Alzheimer's symptoms. In the hippocampus and cerebral cortex, the free-radical scavenging activity of rhoifolin was estimated by calculation of levels for key enzymes (GPx, GRX, SOD and CAT) belonging to the antioxidant defense system. Obtained results showed that rhoifolin could be an promising agent for the management of AD (Huang et al., 2021[76]). 

### Hepatoprotective 

The liver is one of the most important organs in the body that plays an important role in metabolism, detoxification and storage of endogenous and exogenous substances. Due to the vital functions of this organ, liver diseases are among the leading public health problems all over the world. Despite the high level reached by modern medicine, there is not yet a defined drug that supports liver functions, fully protects the organ or helps regenerate liver cells. Thus, it is important to find new pharmaceutical alternatives to be used in the prevention and treatment of liver disorders. Due to the compounds, they contain, some plants play fundamental roles in human health regarding their hepatoprotective potentials that have also been the subject of many scientific studies (Madrigal-Santillan et al., 2014[116]). Alcoholic liver disease (ALD) refers to the spectrum of liver damage due to excessive alcohol consumption (Liu et al., 2019[104]) and its prevalence is increasing worldwide as an important public health problem (Wang and Liu, 2021[186]). *Citrus grandis* is a valuable traditional medicine dating back to hundreds of years in China, and it has been proven to have many pharmacological properties with activity studies. Tea prepared with *C. grandis* is traditionally used to sober up a drunk person due to its hangover-preventing effects. A group that had previously investigated the therapeutic effect of total flavones from *C. grandis* in alcoholic liver disease (Xiao et al., 2012[190]) evaluated the hepatoprotective effects of rhoifolin along with its potential mechanisms. In this study, rhoifolin showed protective effects on a mouse model of ALD and ethanol-treated LO2 cells. Downregulation of CYP2E1, TLR4 expressions, and NF-κB phosphorylation were proposed as the basic mechanisms. Providing an experimental ground for the traditional use of *Citrus grandis*, these results underlined that rhoifolin is a promising herbal remedy for alcoholic liver disease. Additionally, it was also highlighted that further clinical studies should be conducted to confirm the efficacy and safety of this herbal remedy in patients with ALD (Mai et al., 2022[118]).

### Antidiabetic 

Chronic hyperglycemia in diabetic patients leads to an increase in oxidative stress in different tissues and plays an important role in the progression of various related complications including nephropathy, neuropathy, retinopathy, and cardiovascular disorders (Mahmood et al., 2015[117]). The leaves of *Citrus grandis *(L.) Osbeck, also known as red wedun with a high content of rhoifolin (1.1 %, w/w) have been used in Traditional Chinese Medicine (TCM) in the treatment of diabetes. Rhoifolin has the potential to be among the effective precursors for the treatment of diabetes and exerts its antidiabetic effects via increased adiponectin secretion, GLUT4 translocation and phosphorylation of insulin receptor-β in 3T3-L1 fat cells, which explains the traditional use of *C. grandis* in diabetes. Critical genes in the context of the rhoifolin effect may lead to the emergence of new targets in the treatment of diseases that occur as a result of insulin resistance (Rao et al., 2011[144]). 

### Regulatory effect on bone metabolism

Today, the incidence of aseptic loosening after joint-prosthesis replacement operations is increasing. Aseptic loosening can be defined as the failure of joint prostheses without a mechanical cause or any infection. This condition is most often caused by osteolysis (bone resorption) and an inflammatory cellular response in the joint (Hench, 2019[72]). It was revealed that the bone resorption of hyperactive osteoclasts plays a very important role in osteolysis. A study showed that rhoifolin reduced RANKL-induced osteoclastogenesis and bone resorption *in vitro*. In addition, analyses made on titanium particle-induced osteolysis mouse models approved that rhoifolin well ameliorated osteoclast‐stimulated calvarial osteolysis. In this study, it was demonstrated that rhoifolin potently suppressed receptor activators of nuclear factor-κB (NF-κB) ligand-induced osteoclastogenesis, hydroxyapatite resorption, F-actin formation, and expression of osteoclast-related genes. These results especially highlighted the potential of rhoifolin as an important agent for the improvement of prosthesis loosening (Liao et al., 2019[98]).

### Nephroprotective 

Therapeutic use of cisplatin in tumor chemotherapy is limited due to dose-related nephrotoxicity. The effects of rhoifolin on cisplatin-induced nephrotoxicity were investigated by using rats, in which renal damage was demonstrated by decrease in body weight, increase in blood urea, nitrogen and creatinine, and the destruction of histological integrity. Nevertheless, rhoifolin administration ameliorated cisplatin induced nephrotoxicity. Furthermore, rhoifolin administration resulted in alleviation of cisplatin-induced oxidative stress and inflammatory response. Finally, rhoifolin administration inhibited nuclear translocation of NF-κB via downregulation of phospho-IκBα and phospho-p65, and it also up-regulated IκBα, suggesting that it could be a promising adjunct for cisplatin in tumor treatment (Song et al., 2020[167]).

### Cardioprotective

Hypertension is a fairly common condition including various health risks, and studies have mostly focused on cardiovascular diseases and related problems (Buford, 2016[29]). As overexpression of angiotensin-converting enzyme (ACE), which is a fundamental component in the renin-angiotensin-aldosterone system (RAAS) regulating blood pressure, is associated with vascular hypertension, inhibition of ACE is of great importance in respect to hypertension. Recently, research on potential ACE inhibitors started to include natural product derivatives such as peptides, polyphenolics and terpenes (Balasuriya and Rupasinghe, 2011[18]). In a study conducted within this context, 17 flavonoids belonging to five structural subtypes were evaluated *in vitro* for their abilities to inhibit ACE. As a result of this study, which was carried out with two different concentrations (500 µM and 100 µM) using the fluorimetric method, it was determined that the inhibitory potential ranged from 17 % to 95 % at 500 µM and from 0 to 57 % at 100 µM. The IC_50_ value for rhoifolin was determined to be 183 µM. In this study, which showed that flavonoids are an excellent source of functional antihypertensive products, the structural features that increase the activity on the flavonoid skeleton were determined as follows: presence of (a) a catechol group in the B ring, (b) double bond between C2 and C3 in the C-ring, and (c) ketone group at C4 in the C-ring (Guerrero et al., 2012[64]). In another study conducted in the 1990s, acute effects of luteolin, apiin, and rhoifolin on pulmonary vascular circuit were compared with nifedipine in two pulmonary hypertension experimental models during hypoxia and PGF_2α_-induced pulmonary vasoconstriction in dogs under anesthesia. Although 5 mM/kg/ i.v. rhoifolin did not cause any change in hypoxic pulmonary vasoconstriction, it led to decrease in cardiac output and aortic pressure (Occhiuto and Limardi, 1994[128]).

### Antimicrobial 

Antibiotic resistance is an important and growing phenomenon and constitutes a significant public health problem of the 21^st^ century. Though a high number of antibiotics are being used in modern medicine, microbial resistance to these antibiotics has increased significantly due to their inappropriate and widespread usage and the rapid genetic transmission of resistance. The discovery of new drugs that can overcome microbial resistance is of great importance to save humanity from entering a critical era in which minor injuries and infections can become life-threatening (Vadhana et al., 2015[180]). Plants have the potential to provide an unlimited number of antimicrobial compounds due to their rich phytochemical profiles and the fact that they have been used in many different traditional therapeutic applications for centuries (Silva et al., 2013[160]). As one of the largest classes of secondary metabolites that are formed in different parts of the plant, flavonoids exhibit a wide range of pharmacological and beneficial health effects for humans. It is known that plants synthesize flavonoids, especially in response to microbial threats. Scientific studies have shown that these compounds are powerful antimicrobial agents against a variety of pathogenic microorganisms (Gorniak et al., 2019[62]).

Sepsis is a life-threatening organ dysfunction due to microbial infection and is responsible for systemic inflammation leading to organ dysfunction. According to the data reported by the World Health Organization (WHO), mortality due to sepsis is quiet high, about six million deaths occur on a a yearly basis (Hotchkiss et al., 2016[74]). Within the scope of search for alternative treatment methods for this serious health problem, a study on the protective effect of rhoifolin against sepsis was planned. After cecal ligation and puncture method induced sepsis was obtained, rhoifolin was administered to mice at doses of 20 and 40 mg/kg, i.p. for one week. Food intake, survival rates of the mice, and liver function test results and cytokines, were examined. Oxidative stress parameters were also determined in lung tissue homogenate, histopathological analyses were carried out in the liver and lung tissues. The results of the study showed that rhoifolin administration reduced oxidative stress and inflammation in sepsis of the mice via regulation of the TLR4/ MyD88/NF-κB pathway. For this reason, it has been reported that rhoifolin would be beneficial against sepsis and could be used clinically for the management of sepsis (Wen et al., 2023[188]). 

### Antiviral 

COVID-19 pandemic, having severe acute respiratory syndrome coronavirus 2 (SARS-CoV-2) as the causative agent, has become the most important global public health problem in recent years (Popescu et al., 2022[136]). Despite administration of different vaccine types, mutation of new strains and the problems arising from universal immunity have shifted attention to the search for more effective solutions. Flavonoids are the most studied group in terms of antiviral effect, with their success in *in silico, in vitro, in vivo* and recently clinical studies (Kaul et al., 2021[87]). It was reported that the flavonol herbacetin and the flavones rhoifolin and pectolinarin inhibited the enzymatic activity of SARS-CoV 3CLprotease enzyme, which is one of the most studied targets for flavonoid inhibition and these three flavonoids were reported as the best inhibitory compounds against SARS-CoV 3CLpro. They attached to the active sites of proteins and thus inactivated these proteins, resulting in the neutralization of the virus. It has been suggested that hydrophobic aromatic rings and hydrophilic hydroxyl groups are effective in binding affinity. The IC_50_ values of herbacetin, rhoifolin, and pectolinanrin were calculated by the dose-dependent inhibitory curve and found to be 33.17 µM, 27.45 µM, and 37.78 µM, respectively (Jo et al., 2019[82]; Sawikowska, 2020[150]). With regard to SARS-CoV, some studies report that rhoifolin inhibits the 3CL protease enzyme of SARS-CoV (Benarba and Pandiella, 2020[24]; Russo et al, 2020[148]; Abiri et al, 2021[3]; Badshah et al, 2021[17]; Budak et al, 2022[28]; Singh et al, 2022[162]; Sruthi et al, 2023[170]), or show bioactivity; other studies report that it is also bioactive against the main protease and spike glycoprotein of SARS-CoV-2 (Sawikowska, 2020[150]; Tallei et al, 2020[174]; Adhikari et al, 2021[5]; Yantih et al, 2021[196]). Table 6(Tab. 6) (References in Table 6: Abiri et al., 2021[3]; Brinza et al., 2020[27]; Chen et al., 2022[33]; Deng et al., 2022[39]; Eldahshan and Azab, 2012[46]; Guerrero et al., 2012[64]; Huang et al., 2021[76]; Jo et al., 2019[82]; Liao et al., 2019[98]; Long et al., 2023[108]; Mai et al., 2022[118]; Occhiuto and Limardi, 1994[128]; Peng et al., 2020[132]; Rao et al., 2011[144]; Sawikowska, 2020[150]; Song et al., 2020[167]; Wang et al., 2017[184]; Wen et al., 2023[188]; Zhang et al., 2015[203]) summarizes the biological activities of rhoifolin.

## Pharmacokinetic Data and Strategies to Increase the Bioavailability of Rhoifolin

Many flavonoids, especially rhoifolin, having therapeutic efficacy confirmed by *in vitro/in vivo* experiments, have a limited role as therapeutic agents in clinical use due to their physicochemical properties such as low solubility in water or low permeability (Abou Baker, 2022[4]; Ferreira et al., 2022[55]; Maity et al., 2022[119]; Zheng et al., 2022[205]). As a general rule, the pharmacokinetic features of the active substances such as absorption, distribution, metabolism and elimination are mainly affected by their physicochemical properties. In other words, all features of the active molecule such as lipophilicity, solubility in aqueous solutions, molecular weight, size, structure, polar surface area, ability to form hydrogen bonds, and resistance to enzymatic reactions are effective parameters for the bioavailability of the active ingredient (Smith, 1997[164]; Loftsson, 2015[107]). On the other hand, unpleasant odor or taste; stability problems caused by many factors such as light or heat during the storage stage, as well as body pH differences may also result in less or no therapeutic effect of the active ingredient. For example, quercetin has very limited clinical use due to its low water solubility, while epigallocatechin gallate is known to be unstable in neutral or alkaline solutions, and naringin is poorly absorbed when administered orally (Barras et al., 2009[20]). Hence modern drug delivery strategies with different methods and processes can come into play to overcome the physicochemical drawbacks of phytochemicals and to convert a biologically active compound into a therapeutically effective drug (Puglia et al., 2017[138]). Phytochemicals can be found on the market as dietary supplements, conventional drugs or cosmetics. Nanotechnology-based carriers, called colloidal drug carrier systems, are one of the most up-to-date approaches for biopharmaceutical applications of phytochemicals since they provide a solution for an active molecule which is insufficient in treatment alone. Colloidal drug delivery systems including polymeric nanoparticles, lipidic nanoparticles, liposomes, niosomes or phytosomes etc. are particles and vesicles with three external nanoscale dimensions. They should have a size range of about 1 to 1000 nm. Due to the unique and tunable size-dependent properties of colloidal drug delivery systems and their ability to trap phytochemicals in nanocarriers, both increased efficacy and stability can be achieved while offering controlled and site-specific drug delivery. When an active molecule, in this case, flavonoids, is embedded or entrapped in the matrix of nanocarriers, controlled and sustained release profiles can be obtained and prolongation of the systemic circulation lifetime of the molecule can be achieved. In addition, significant improvements in the pharmacokinetics and therapeutic index of the flavonoid can be achieved in the presence of a colloidal carrier system. One of the effective parameters here is particle size and so the increased surface area.

The colloidal size causes an increase in interaction with the biological environment and provides ease of overcoming many biological barriers. Furthermore, the safety and effectiveness of the phytochemical can be increased by reducing the dose and thus its side effects. By another way of explanation, colloidal drug delivery systems have been developed for the purpose of obtaining maximum therapeutic efficiency with minimum side effects for phytochemicals that are insufficient in treatment alone. Both topical and systemic applications such as oral, nasal, dermal, parenteral or ocular routes can be used for the bioactive molecule. As a result, colloidal carrier systems and nanomedicine offer a new perspective on therapeutic quality for various flavonoids, especially rhoifolin, as well as patient compliance (Jeevanandam et al., 2018[81]). Rhoifolin is a type of flavone glycoside belonging to the apigenin family known also as “apigenin 7-*O*-neohesperidoside” and preclinical studies have demonstrated that rhoifolin exhibits many potent therapeutical activities such as anti-inflammatory, antioxidant, antibacterial, antiviral (Eldahshan, 2013[45], Negm et al., 2022[125]); anticancer (Eldahshan, 2013[45], Zheng et al., 2022[205]); antidiabetic (Brinza et al., 2020[27]); hepatoprotective (Refaat et al., 2015[146]); antirheumatic (Peng et al., 2020[132]) properties. Despite the potential therapeutic efficacy of rhoifolin or similar phenolic compounds, their easy degradation by environmental stress and low water solubility are the main limitations of their application as drugs. Considering the aforementioned limitations, many colloidal drug delivery system studies have been carried out on fully characterized flavonoids such as apigenin and naringin. In a recent study, rhoifolin isolated from Jordanian *Teucrium polium*, then rhoifolin-loaded poly(lactide-co-glycolide)-PLGA nanoparticles were obtained by single emulsion (O/W) solvent evaporation technique (Al-Shalabi et al., 2022[10]). Optimum polymeric nanoparticle formulation was selected by evaluating the loading capacity results after pharmaceutical development studies and tannic acid-mediated surface modification was carried out with poly(ethylene glycol)-PEG. While optimum rhoifolin-loaded PLGA nanoparticles have a particle size of 182 ± 8 0 nm, a PDI value of 0.15 ± 0.01, −27 ± 8 mV surface charge, and an encapsulation efficiency percentage of 45.0 ± 4.3 %, after PEGylation process; particle size, PDI and zeta potential were measured as 204 ± 2 nm, 0.14 ± 0.02 and −28 ± 3 mV, respectively. One of the most important data obtained in this study is that the surface modification process of rhoifolin-loaded PLGA nanoparticles with hydrophilic polymer-PEG does not cause a statistically significant change in the physicochemical properties of these nanoparticles. It has been emphasized that the advantage of the PEGylation process is to increase the water solubility of rhoifolin-loaded polymeric nanoparticles, as well as to prolong their systemic circulation, reduce their immunegenicity and reduce their accumulation in reticuloendothelial system. On the other hand, surface modified nanoparticles maintained their colloidal stability both during the storage stage and in the presence of serum. Another remarkable finding obtained in the study was rhoifolin nanoparticles' antioxidant and free radical scavenging capacity. Cell-based assay with RAW 264.7 murine macrophage cells as well as the *in vitro *cell-free ABTS assay results show that it exhibits enhanced antioxidant activity and cellular uptake when bioactive flavonoid is entrapped into nanoparticles. *In vivo* efficacy tests with *in vivo* paw edema test and histopathological studies in rats confirmed the role of rhoifolin-loaded polymeric nanoparticles in alleviating paw edema and also confirmed that both free rhoifolin and rhoifolin-loaded polymeric nanoparticles possessed potent anti-inflammatory activity.

## Limitations

The current research on the anticancer properties of rhoifolin, while promising, is hindered by several significant limitations and clinical gaps that must be addressed to facilitate its advancement into clinical use. Primarily, the majority of studies have been conducted *in vitro* or in animal models, with a noticeable lack of clinical trials evaluating rhoifolin's safety, efficacy, and pharmacokinetics in human subjects. This absence of clinical data significantly impedes the translation of preclinical findings into practical therapeutic applications. Additionally, rhoifolin's poor water solubility and low bioavailability present substantial challenges for its clinical application. Although recent developments in nanotechnology-based delivery systems have shown potential in enhancing its bioavailability, further research is necessary to optimize these systems and validate their effectiveness in humans. Moreover, the variability in rhoifolin concentration depending on plant sources and environmental conditions poses challenges for the standardization of its extracts, which is fundamental for consistent therapeutic outcomes. The current understanding of the molecular mechanisms underlying rhoifolin's anticancer activity remains incomplete, with only a partial elucidation of its pathways, limiting the ability to develop targeted and effective therapies. Furthermore, there is a lack of comprehensive toxicological studies, which are essential to determine the safe dosage ranges and potential side effects of rhoifolin, particularly for its use in long-term cancer treatments. Another significant gap is the limited exploration of rhoifolin in combination with other anticancer agents, which could potentially enhance or synergize with existing treatments, offering new therapeutic strategies. Finally, the development of rhoifolin as a viable therapeutic agent faces regulatory challenges due to the limited clinical data and the need for standardized formulations, which necessitates coordinated efforts between researchers, clinicians, and regulatory authorities. Addressing these limitations through rigorous research and clinical trials is essential to fully realize the therapeutic potential of rhoifolin in oncology.

## Conclusion

Rhoifolin, apigenin-7-*O*-neohesperidoside is a flavonoid derivative that is found in many plant species belonging to various plant families. Within these plant species, we can see that the compound is not limited to certain plant parts, and thus, can be found in every part of different species such as seeds, flowers, roots, leaves, fruits, and stems. Having been found in hundreds of plant species in varying quantities, this flavonoid has important bioactivities which deserve more research, especially related to its anticancer activity since novel compounds with low side effects are constantly being sought for a couple of decades. While rhoifolin is an important candidate for the development of anticancer drugs, its low bioavailability due to low water solubility and easy degradation by environmental stress limits its usage for medicinal purposes. This difficulty might be overcome in the future with the help of biotechnology studies performed with nanoparticles in which the compound could be encapsulated in, thus more studies related to anticancer activity focusing on increasing the bioavailability of rhoifolin are needed.

## Notes

Jesús Herrera-Bravo, Javad Sharifi-Rad (Universidad Espíritu Santo, Samborondón 092301, Ecuador; E-mail: javad.sharifirad@gmail.com) and Daniela Calina (Department of Clinical Pharmacy, University of Medicine and Pharmacy of Craiova, 200349 Craiova, Romania; E-mail: calinadaniela@gmail.com) contributed equally as corresponding author.

## Declaration

### Competing interests

The authors wish to confirm that there are no known conflicts of interest associated with this publication and there has been no significant financial support for this work that could have influenced its outcome.

### Funding

Not applicable.

## Figures and Tables

**Table 1:**
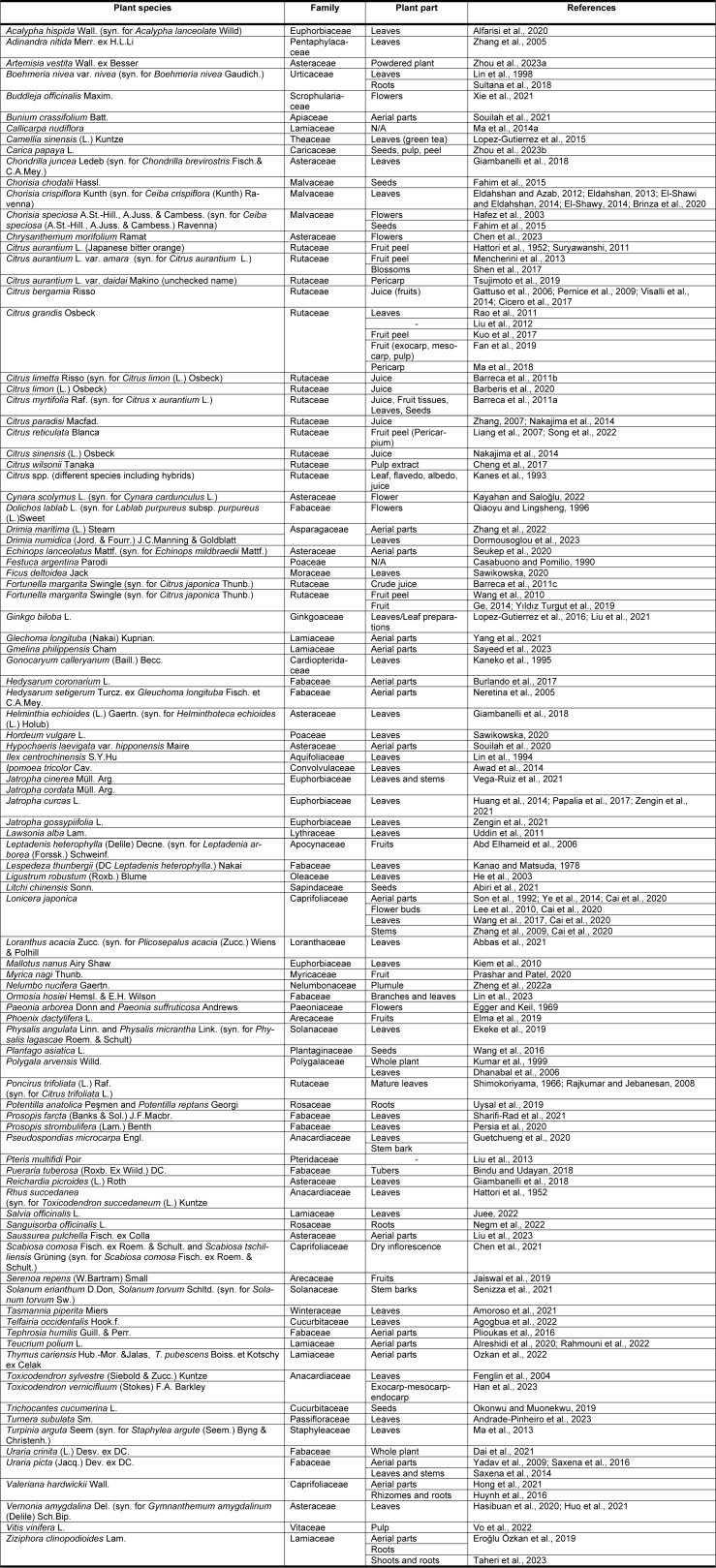
Plant species that are reported to contain rhoifolin

**Table 2:**
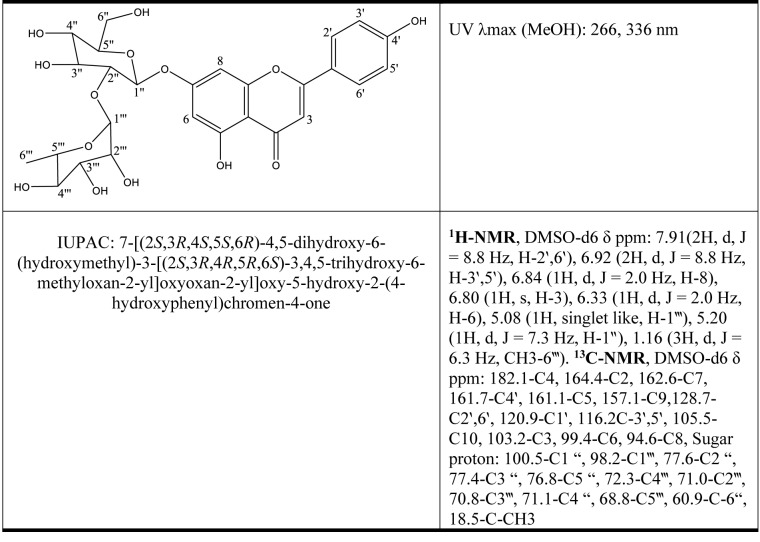
Chemical structure, nomenclature, UV absorbance and NMR spectral data of rhoifolin

**Table 3:**
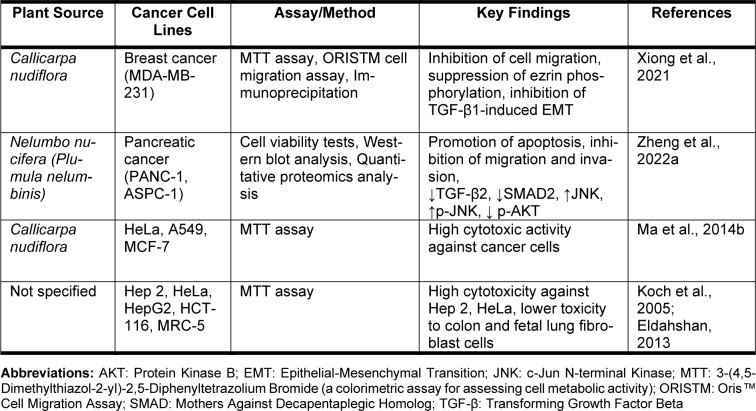
Summary of *in vitro* anticancer activities of rhoifolin

**Table 4:**
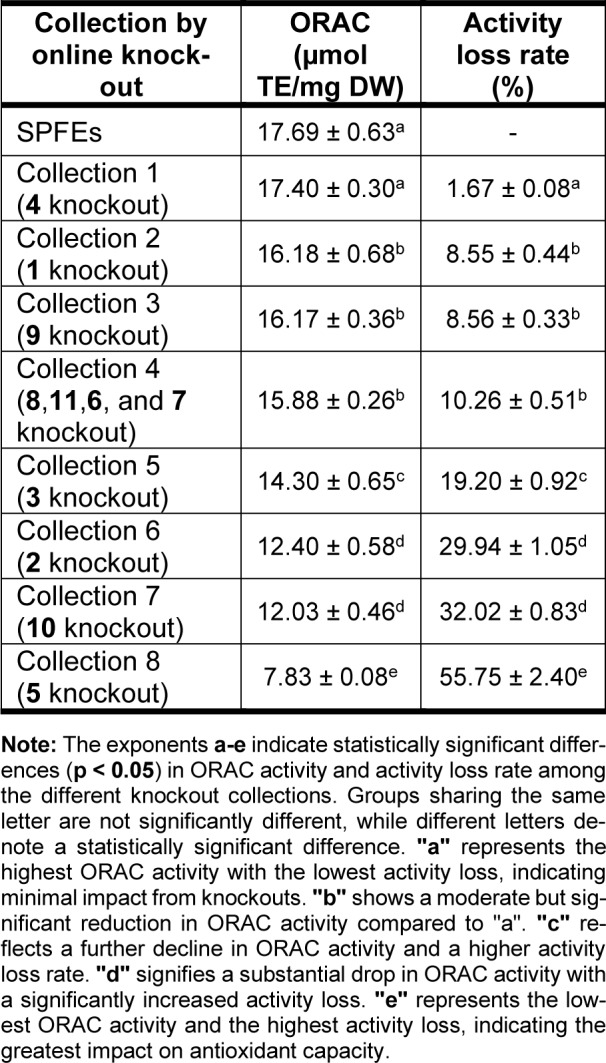
ORAC activity of SPFEs and targeted knockout collections of one or more isolated flavonoids and the activity loss rate values gained according to the knockouts. Values with different letters in each column indicate significant differences (p < 0.05). The mg DW in the unit of ORAC refers to the weight of SPFEs (adapted from Deng et al., 2022).

**Table 5:**
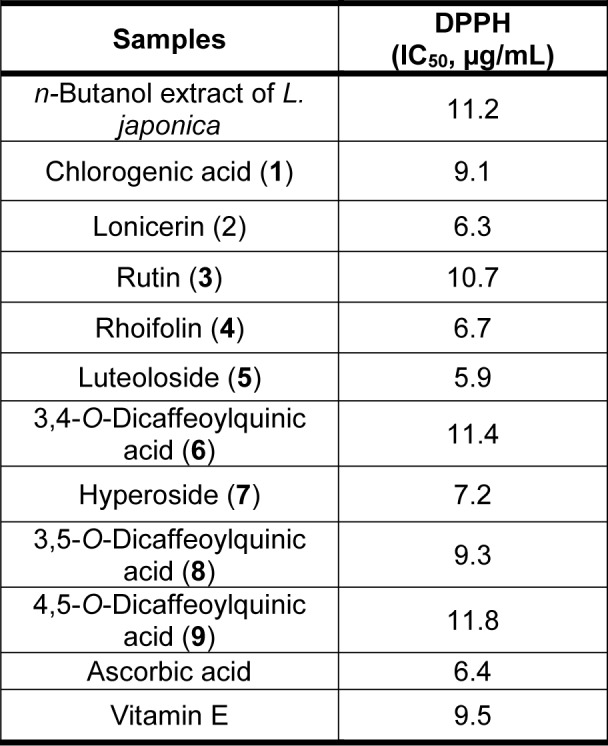
Antioxidant activity values of crude extracts and the isolated compounds from *L. japonica *leaves in DPPH assay (adapted from Wang et al., 2017)

**Table 6:**
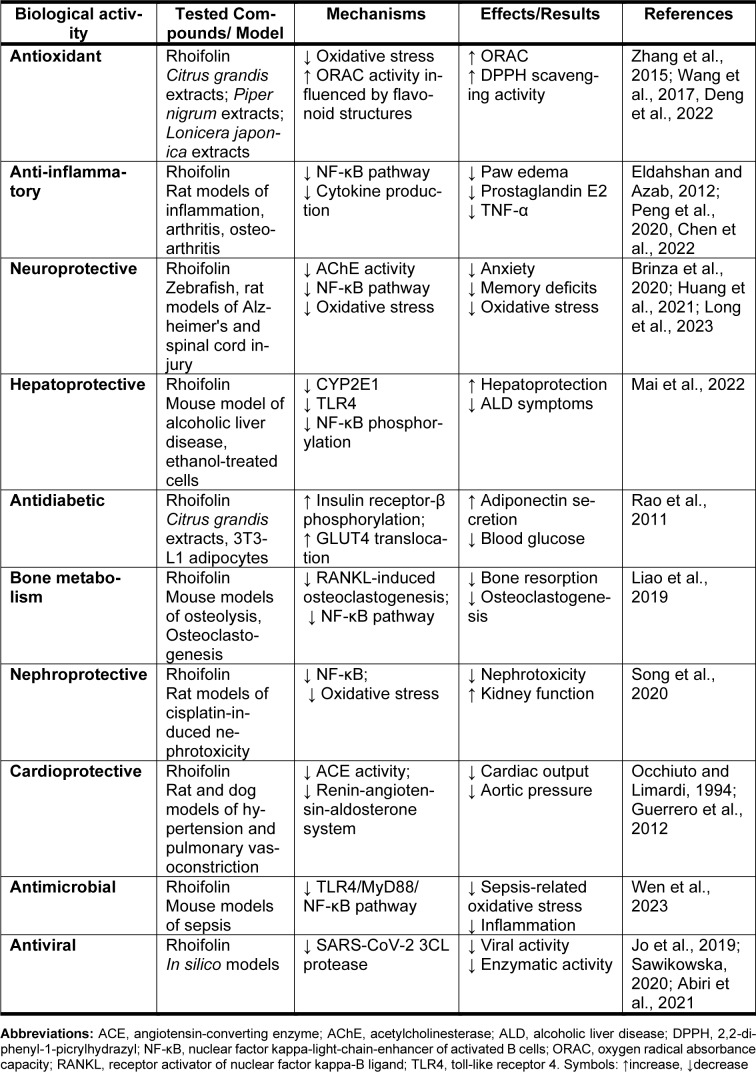
Other pharmacological properties of rhoifolin
